# Clap-and-fling mechanism in a hovering insect-like two-winged flapping-wing micro air vehicle

**DOI:** 10.1098/rsos.160746

**Published:** 2016-12-07

**Authors:** Hoang Vu Phan, Thi Kim Loan Au, Hoon Cheol Park

**Affiliations:** 1 Artificial Muscle Research Center, Konkuk University, Seoul 143-701, South Korea; 2 Department of Advanced Technology Fusion, Konkuk University, Seoul 143-701, South Korea

**Keywords:** clap and fling, two-winged flapping-wing micro air vehicle, biomimetics, rhinoceros beetle, insect flight

## Abstract

This study used numerical and experimental approaches to investigate the role played by the clap-and-fling mechanism in enhancing force generation in hovering insect-like two-winged flapping-wing micro air vehicle (FW-MAV). The flapping mechanism was designed to symmetrically flap wings at a high flapping amplitude of approximately 192°. The clap-and-fling mechanisms were thereby implemented at both dorsal and ventral stroke reversals. A computational fluid dynamic (CFD) model was constructed based on three-dimensional wing kinematics to estimate the force generation, which was validated by the measured forces using a 6-axis load cell. The computed forces proved that the CFD model provided reasonable estimation with differences less than 8%, when compared with the measured forces. The measurement indicated that the clap and flings at both the stroke reversals augmented the average vertical force by 16.2% when compared with the force without the clap-and-fling effect. In the CFD simulation, the clap and flings enhanced the vertical force by 11.5% and horizontal drag force by 18.4%. The observations indicated that both the fling and the clap contributed to the augmented vertical force by 62.6% and 37.4%, respectively, and to the augmented horizontal drag force by 71.7% and 28.3%, respectively. The flow structures suggested that a strong downwash was expelled from the opening gap between the trailing edges during the fling as well as the clap at each stroke reversal. In addition to the fling phases, the influx of air into the low-pressure region between the wings from the leading edges also significantly contributed to augmentation of the vertical force. The study conducted for high Reynolds numbers also confirmed that the effect of the clap and fling was insignificant when the minimum distance between the two wings exceeded 1.2c (c = wing chord). Thus, the clap and flings were successfully implemented in the FW-MAV, and there was a significant improvement in the vertical force.

## 1. Introduction

The flight of insects is a source of inspiration for several researchers in robotics because of their potential application in the development of flapping-wing micro air vehicles (FW-MAVs) [1–6]. Various studies on insect wings explored the basic principles of complex and unsteady force generation mechanisms during flight such as the clap-and-fling [7–12] effect, leading edge vortex (LEV) generation [9,10,13–15], delayed stall of the LEV, and wake capture and rotational circulation [15,16]. In contrast with the other mechanisms, the clap-and-fling effect is not considered as a typical method of lift generation in insect flight. The clap and fling occurs due to the interaction between two flapping wings at dorsal stroke reversal, and functions to improve the lift generation. It was first discovered by Weis-Fogh [7] based on the captured hovering wing kinematics of the tiny *Encarsia formosa* wasp. The clap is placed when the leading edges of the left and right wings approach each other, prior to when the trailing edges of the wings approach each other at the end of upstroke. Following the clap, the wings commence the fling phase consisting of the next downstroke motions by rotating the wings about their trailing edges and moving the leading edges apart from each other [9]. Most tiny insects such as wasps [7,12], diptera [17,18], lacewings [19], whiteflies [20,21] and thrips [22,23] use the clap-and-fling mechanism frequently during flight. However, this mechanism is not frequently used in larger insect species except during take-off, or when carrying a load [24] or performing power intensive manoeuvres [25]. In insects with flexible wings, the clap and fling is referred to as a clap-and-peel mechanism because the fling and the clap function in a manner similar to a peel and a reverse peel, respectively [17]. This can be observed in Drosophila [26], butterflies [27–29], bush cricket, mantis [30,31] and locusts [25]. Additionally, observations on white butterflies (*Pieris barssicae*), bluebottles (*Calliphora vicina*) and flour moths (*Ephista*) revealed that their left and right wings approach each other partially without touching the wings at the dorsal stroke reversal and this presents a near-clap-and-fling pattern [17,18,32].

Experimental and computational studies have extensively investigated the effect of the clap and fling on the aerodynamic lift generation in insect flight [12,24,33–37]. Miller & Peskin [12] used the immersed boundary method for a low Reynolds number of 10 to investigate the effect of wing flexibility on aerodynamic performance during a clap-and-fling process. The study indicated that the clap-and-fling mechanism in the flexible wing reduced the drag by approximately 50%, while relatively improving the lift when compared with those in a rigid wing. Bennett [33] conducted two-dimensional experiments at a Reynolds number of 83 000 by using a robotic rectangular wing with a vertical wall as a symmetric plane to observe the benefits of the clap-and-fling effect. The measurement indicated that the wing in the presence of vertical plane contributed to an increase of 15% in the total lift when compared with that in the absence of vertical plane. An experimental study on various insects, small birds and bats conducted by Marden [24] reported that flapping wings with the clap-and-fling effect led to an increased lift per unit flight muscle mass of approximately 25% when compared with that of conventional flapping wings without the clap-and-fling motion. Furthermore, a numerical study by Sun & Yu [36] performed a two-dimensional simulation at a Reynolds number of 17 and revealed that the clap-and-fling effect augmented the lift generation when compared with that of a single wing. Studies also examined the effect of the distance between the hinges of two wings on enhancing lift and torque generations. An increase in the distance from 0.1c to 0.2c (c = wing chord) resulted in a slight decrease in lift but greatly attenuated detrimental torque [36]. The lift and torque enhancements were diminished when the distance approached 1c [36]. Lehmann *et al*. [37] performed experiments based on the small three-dimensional wing of a *Drosophila* fruit fly at Reynolds numbers of 100–200, and found that the near-clap-and-fling effect could lead to a lift enhancement of 17% based on wing kinematics.

The clap-and-fling mechanism in insect flight was extended and applied in several four-winged FW-MAVs to improve the lift generation [4,38,39]. Groen *et al*. [40] investigated the effect of the clap and peel on thrust generation in a Delfly FW-MAV and revealed that due to the peel at the beginning of the strokes there was a gain of only 6%. However, the experiments on the Mentor FW-MAV showed that the clap-and-fling effect significantly increased the thrust and the thrust-to-power ratio by approximately 50% and 40%, respectively [38]. Nguyen *et al*. [41] also obtained an improvement in the lift generation of an FW-MAV, which combined two flapping wings and two fixed wings by implementing the clap-and-fling effect. Their experimental results showed that the dorsal and ventral clap and flings contributed to an enhanced lift of approximately 45% when compared with that in the non-clap-and-fling case. Thus, the clap-and-fling effect played an important role in improving the lift of FW-MAVs.

Most available FW-MAVs use a four-winged mechanism to implement the clap and flings at the stroke reversals instead of the two-winged mechanism used in insects. This is mainly because a large flapping angle exceeding 180° is required to implement the clap-and-fling effect with two wings. As a result, the flapping amplitude of each wing is relatively smaller than that in insect flight. A study by De Clercq *et al*. [42] showed that only the fling augmented the force generation. Most studies based on an experimental approach could not identify the contribution of each phase, i.e. ‘clap’ and ‘fling’ to the force enhancement [38,41]. Therefore, the effect of the clap was not clearly discussed in the extant literature. Moreover, studies on insects indicated that the unexpected drag force produced by the clap-and-fling effect exceeded that of the single wing at low Reynolds numbers [11,12]. The drag force could be significantly reduced by using the flexible clap and fling. However, its magnitude was still approximately five times that of the magnitude of the drag force in the single-winged case [12]. However, the effect of the clap and fling or clap and peel on the drag force was not considered in the above FW-MAVs, which involved flapping wings at high Reynolds numbers.

This study proposed a hovering insect-like two-winged FW-MAV, which integrated the clap-and-fling mechanism at each stroke reversal in an effort to mimic the flight of a hovering *Allomyrina dichotoma* or rhinoceros beetle. In order to create a high flapping amplitude, the flapping mechanism was designed by using a combination of four-bar linkage and pulley–string mechanism. The contribution of the clap and fling at a high Reynolds number of 15 000 to the force generation was investigated by both computational and experimental approaches. The three-dimensional flapping-wing kinematics was first obtained by conducting a measurement using three synchronized high-speed cameras. Then, the computational fluid dynamic (CFD) was performed based on the measured three-dimensional wing kinematics to estimate the force generation and flow structures produced by the wings with and without the effect of the clap and fling during hover. The forces generated by the FW-MAV were measured using a load cell and the measured forces were compared with those obtained from the CFD.

## 2. Observation of beetle flight

**Figure 1.**
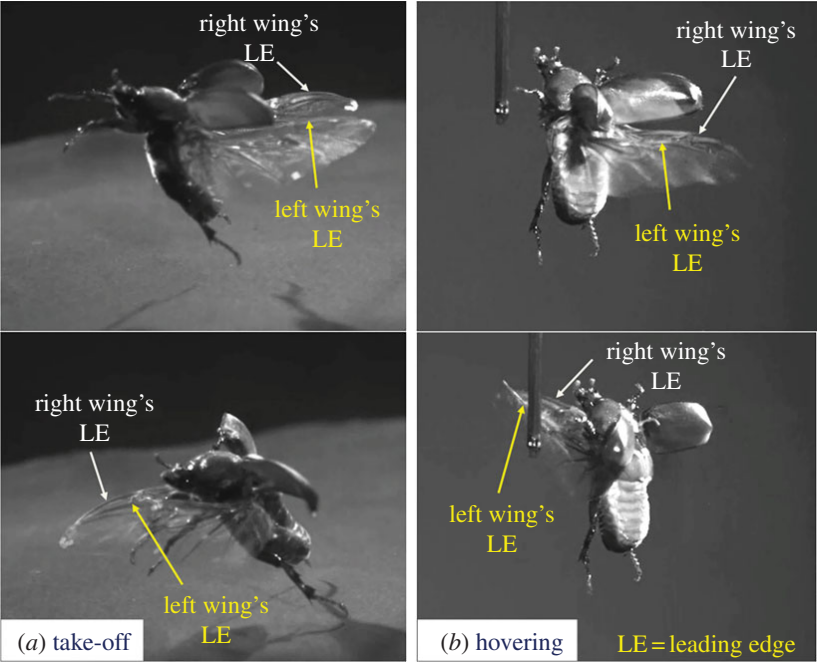
Snapshot of the kinematics of a rhinoceros beetle's hind wing at the ends of the upstroke and downstroke during (*a*) take-off and (*b*) hovering.

A rhinoceros beetle or *A. dichotoma*, with an approximately weight in the range of 5–10 g [43,44], is among the largest flying insects that can perform hovering during flight. The ranges of the Reynolds number and flapping frequency of this particular beetle are 10 000–15 000 and 35–40 Hz, respectively [43,44]. Additionally, the beetle is capable of flapping its hind wings with a very high flapping amplitude [43–46]. The flapping amplitude of the hind wing is approximately 165 ± 5° during forward flight at a velocity of 1.5 m s^−1^ with a stroke plane angle or the angle between the stroke plane and the horizontal plane of approximately 72° [45]. At a lower forward velocity of 0.44 m s^−1^ and stroke plane angle of approximately 30°, the flapping amplitude increases to approximately 180 ± 5° [47]. The hind wing's flapping amplitude can equal or exceed 180° during take-off and hovering when the stroke plane is nearly parallel to the horizontal plane [43,46]. The effect of clap and fling has not yet been studied for this type of beetle. Nevertheless, the high flapping amplitude of the hind wing during the flapping motion indicates a possible use of the clap and flings at the dorsal and ventral stroke reversals. The beetle increases the flapping angle until the two wings touch each other to enhance lift, particularly during take-off (figure 1*a*), hovering (figure 1*b*) and even during the action of carrying a load. This increase in the flapping amplitude may result in the appearance of the clap-and-fling effect [24]. Furthermore, the beetle's hind wing can also perform a spanwise twist and chordwise camber during the flapping motion [44,45]. Le *et al*. ([45], fig. 4(c,d)) showed that the hind wing twisted linearly from the wing root to the wing tip and created a chordwise camber of less than 20% wing chord. Several studies suggested that these wing deformations could improve the flight performance of the insect [45,48–51]. Thus, mimicking the above-mentioned features of the beetle's hind wing could be useful in improving the force generation of an FW-MAV.

## 3. Material and methods

### 3.1. Flapping mechanism

**Figure 2.**
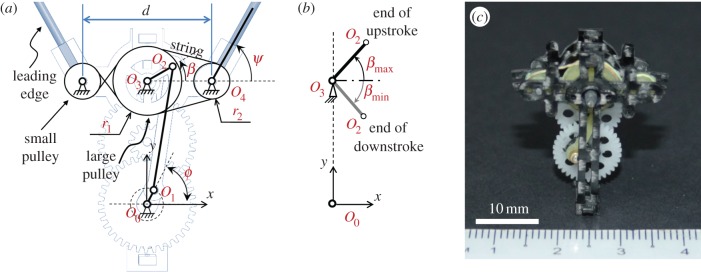
(*a*) Schematic of the flapping mechanism, (*b*) definition of maximum and minimum values of angle *β* (*β*_min_* *=* *−*β*_max_) and (*c*) the fabricated flapping mechanism without wings.

A flapping mechanism based on a combination of four-bar linkage and pulley–string mechanism was designed for high flapping amplitudes to mimic the wing motion of the rhinoceros beetle. Figure 2*a* illustrates the schematics of the flapping mechanism. The rotary motion of the crank *O*_1_*O*_2_ is converted to the flapping motion of the linkage *O*_2_*O*_3_ through the couplers *O*_1_*O*_2_, which are rigidly glued to the large pulley. A small pulley is connected to the large pulley through a string to amplify the flapping motion (*β*) of the linkage *O*_2_*O*_3_ to a larger flapping motion of the output link (*ψ*), which is glued to the small pulley. An end of the leading edge of the wing is connected to the output link to create the flapping motion. The connecting string between the large pulley and small pulley at one side is twisted to create the flapping motions of two small pulleys moving in the same direction to create flapping-wing motions in the two wings. Figure 2*b* defines the maximum and minimum values of the sweeping angle *β*, whose magnitudes are identical. When the *O*_0_*O*_3_, *O*_2_*O*_3_, *β*_max_ and *β*_min_ are predetermined as input parameters of the design, the length of linkages can be expressed as follows:

3.1 $$O_{0}O_{1}=\frac{1}{2}\left(\sqrt{O_{0}O_{3}^{2}+O_{2}O_{3}^{2}+2O_{0}O_{3}.O_{2}O_{3}\sin\;\beta_{\max}}-\sqrt{O_{0}O_{3}^{2}+O_{2}O_{3}^{2}+2O_{0}O_{3}.O_{2}O_{3}\sin\;\beta_{\min}}\right)$$

and

3.2 $$O_{1}O_{2}=\frac{1}{2}\left(\sqrt{O_{0}O_{3}^{2}+O_{2}O_{3}^{2}+2O_{0}O_{3}.O_{2}O_{3}\sin\beta_{\max}}+\sqrt{O_{0}O_{3}^{2}+O_{2}O_{3}^{2}+2O_{0}O_{3}.O_{2}O_{3}\sin\beta_{\min}}\right).$$

The relationship between the flapping angle *ψ* of the small pulley and the flapping angle *β* of the large pulley can be determined as follows:

3.3 $$\psi =\frac{r_{1}}{r_{2}}\beta .$$

Carbon/epoxy panels with a thickness of 0.8 mm were used to fabricate all linkages and supporting frames. The parts were built with a CAD design software by using a CNC machine (MM-300S, resolution 10 µm, MANIX, Korea), and then manually assembled as shown in figure 2*c*. A reduction gear ratio of 21 : 1 was selected in the flapping mechanism to amplify the output torque from a DC motor.

### 3.2. Wing design and wing kinematics

**Figure 3.**
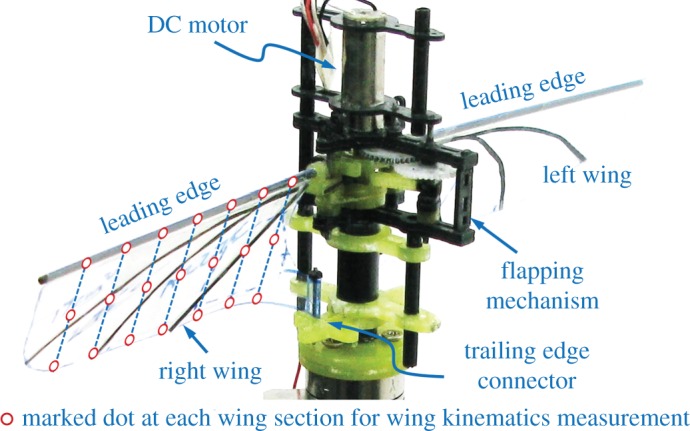
Wing configuration used to create wing twist and camber. The wing membranes are freely rotated about the leading edges, while the wing roots at the trailing edges are clamped to the wing root connector.

The wing was composed of veins made of carbon strips and thin membranes made of polyethylene terephthalate. The wingspan from the wing root to the tip (*R*) was approximately 70 mm and the wing mean chord $\left(\bar{c}\right)$ was approximately 25 mm. The leading edges of the wings made of carbon rod with a diameter of 1.2 mm were attached to the output links of the flapping mechanism, while the wing roots were connected to the trailing edge connector, as shown in figure 3. The wing membrane was designed to freely rotate around the carbon rod at the leading edge. With this configuration, the flapping-wing system could produce the passive spanwise twist and chordwise camber during the flapping motion. More details can be found in Phan *et al.* [52].

Details on the measurements of the wing kinematics can be found in previous studies [45,47] as this study only provides a brief summary of the measurement process. White dots were marked on the wing along seven wing chords located at 12.5%*R*, 25%*R*, 37.5%*R*, 50%*R*, 62.5%*R*, 75%*R* and 87.5%*R*, as shown in figure 3. Three high-speed cameras synchronized at 2000 fps with a resolution of 1024 × 1024 pixels were used to track the locations of the marked dots on the wing during the flapping motion. Then, the three-dimensional coordinates of the marked dots were determined by analysing the sequential images obtained from the high-speed cameras by using the direct linear transformation method developed in a Matlab code [53].

The time histories of the flapping angle and the rotation angles were then obtained from the coordinates of the dots during the flapping motion. The first eight terms of sine and cosine functions were added, and the summation was used as a fitting function to fit the measured time histories of the flapping angle and wing rotation angles, based on the least-square method [54] as expressed below:

3.4 $$\kappa (t)=\sum_{k=0}^{8}\left[a_{k}\cos(2k\pi ft\right)+b_{k}\sin(2k\pi ft)],$$

where *κ*(*t*) denotes the fitted flapping angle or rotation angle at an instant time *t*, *f* denotes the flapping frequency and *a*_0_, *a_k_* and *b_k_* denote the fitted coefficients.

### 3.3. Computational method

The three-dimensional deformable wing kinematics was described as an input condition in the commercial CFD solver (Fluent 16.2 package) to estimate the force generation and flow structure around the wings during the flapping motion. The wing motion was defined by using a user-defined function at a flapping frequency of 20 Hz to obtain a Reynolds number of approximately 15 000. Young & Lai [55] used a dynamic mesh feature for a turbulent model, which was found to have no difference with the laminar model in terms of the force generation of a flapping wing at Reynolds numbers ranging from 100 to 50 000. Therefore, in this study, an incompressible laminar model was chosen to simulate the airflow around the wing. Similar CFD with laminar model was explored in previous studies [49,56,57].

**Figure 4.**
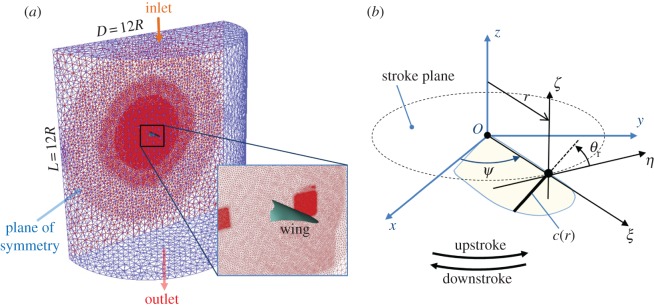
(*a*) Computational domain for CFD calculation and (*b*) definition of wing motion.

Only one wing was simulated with a symmetric condition, as the flapping mechanism was designed to flap the wings symmetrically in a symmetric plane as shown in figure 4. The computational domain included a half cylinder with a diameter (*D*) and a length (*L*) of 12*R* (840 mm), as show in figure 4*a*. The wing was placed behind the inlet at a distance of 6*R* (420 mm), and the wing surface was considered as a membrane without any roughness. In the hovering condition, there was no inflow velocity at the inlet. Six flapping cycles were simulated at a time step of 1/1000 of a cycle. The motion of a wing was a combination of flapping around the flapping axis (*z*-axis) and rotation around the feather axis (*ξ*-axis), which was attached to the leading edge of the wing, as shown in figure 4*b*. The flapping angle, denoted by *ψ*, was defined as the angle between the *x*-axis and the feather axis. The rotation angle, denoted by *θ*_r_, was determined by the angle between the *η*-axis and the wing chord. The distance from the flapping axis to the symmetric plane was 8 mm (*d*/2 = 8 mm), which is equal to half the distance between the flapping axes of the two wings. In order to investigate the effect of the clap and fling, the forces generated in this case were compared with those in the other case where the distance between the flapping axis and the symmetric plane extended to 20 mm (*d*/2 = 20 mm), which was sufficiently far to eliminate the clap-and-fling effect. Henceforth, the other case is referred to as the non-clap-and-fling case, in this study.

### 3.4. Experimental method

**Figure 5.**
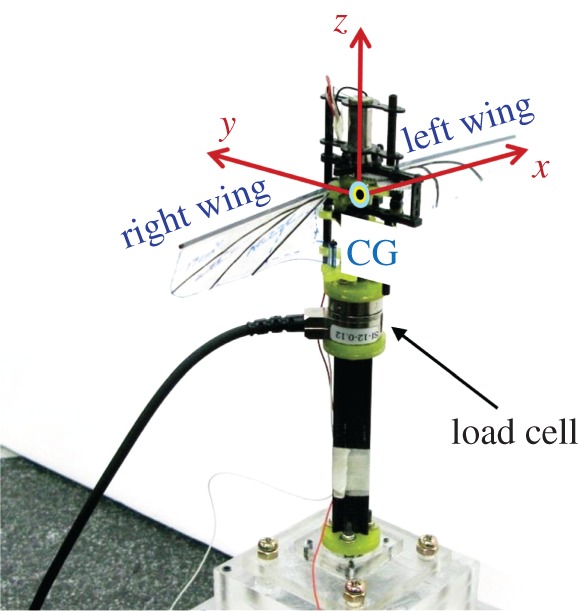
Set-up for force measurement using a 6-axis load cell.

The forces generated by the flapping wings were measured by using a 6-axis load cell (Nano 17, Stainless steel, ATI Industrial Automation, USA, force resolution of 2.94 mN) as shown in figure 5. The flapping-wing system was excited by an external power supply (E3646A, Agilent, Malaysia) at the same flapping frequency of 20 Hz as that applied to the CFD model. The flapping-wing system was operated for approximately 100 flapping cycles in each test. The measured forces acquired from more than 10 experiments were averaged.

**Figure 6.**
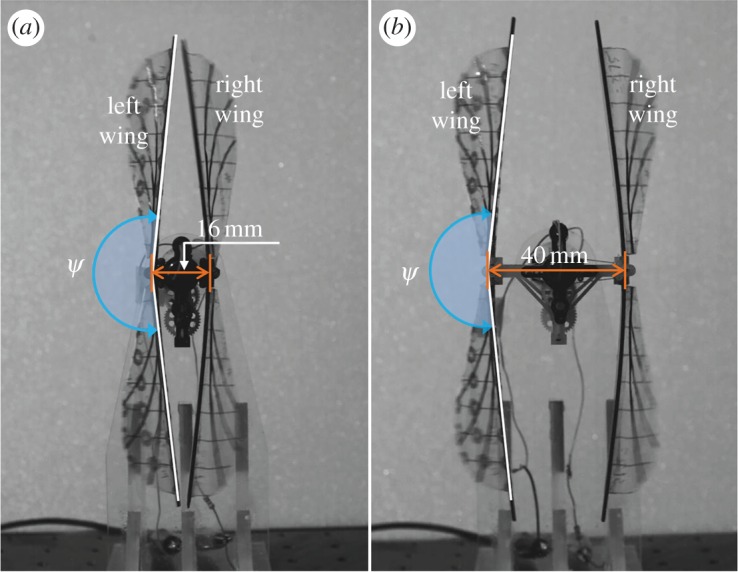
Composite images of two flapping-wing models used for the force measurement. (*a*) The flapping-wing system with the clap-and-fling effect placed at each stroke reversal and (*b*) the flapping-wing system with an extended distance between the flapping axes to minimize the effect of the clap and fling.

A flapping-wing system was fabricated with an extended distance between two flapping axes, as shown in figure 6, to investigate the manner in which the forces changed without the effect of the clap and fling at the stroke reversals. All other design parameters in this model were theoretically the same as those in the flapping-wing system with the clap-and-fling effect. The only difference was the distance between two flapping axes of the two wings, which was extended to 40 mm or 1.6c. A study by Sun & Yu [36] indicated that this distance was sufficiently far to minimize the interaction effect of the wings at each stroke reversal. The flapping-wing system was also installed in the load cell for force measurement at a frequency of 20 Hz, and compared in terms of force generation with the flapping-wing system with the clap-and-fling effect. The time history of the force generation during the flapping motion was obtained by filtering the raw data using a low-pass filter with a cut-off frequency that was five times higher than the flapping frequency to eliminate the high-frequency effect from noises and structure vibrations.

## 4. Results and discussion

### 4.1. Wing kinematics

**Figure 7.**
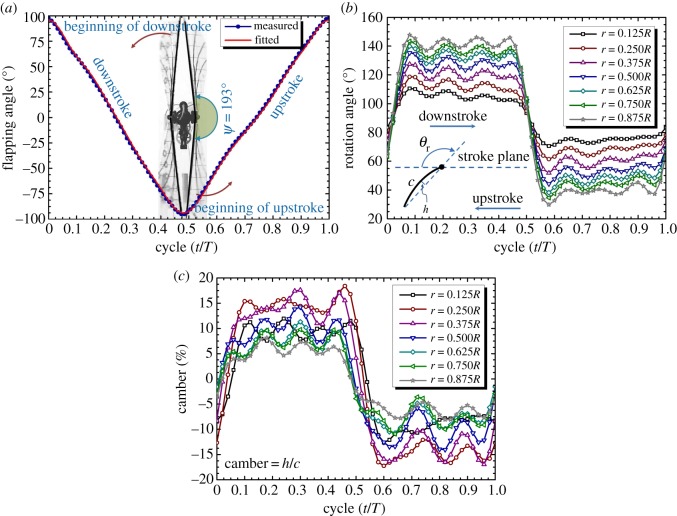
Time histories of (*a*) flapping angle, (*b*) wing rotation angle and (*c*) camber deformation at different wing sections measured along the wingspan during the flapping motion.

Figure 7*a* shows a plot of the time history of the flapping angle of the leading edge. The wing commenced a flapping cycle from the beginning to the end of the downstroke in the period (*t*/*T*) from 0 to approximately 0.50. Following this, the wing began the upstroke in the period from approximately 0.50 to 1.00. The measured peak-to-peak values of the flapping angle ranged from approximately 97.2° to −95°, and thus the measured flapping amplitude was approximately 192.2°. In order to precisely track the time history of the measured flapping angle, 8-term sine and cosine functions were used as a fitting function for the CFD model inputs. The fitted values of the flapping angle at the end of each stroke were approximately 95.6° and −93.5°, and the flapping amplitude was 189.1°. Hence, the fitted amplitude was approximately 3.1° smaller than the measured angle, and this is an acceptable error. Figure 7*b* shows the fitted wing rotation angles at seven wing sections using 8-term sine and cosine functions. The variation of the rotation angle indicated that the wing was twisted from the wing root to the wing tip during the translational phase (0.10 ≤ *t*/*T* ≤ 0.45 and 0.60 ≤ *t*/*T* ≤ 0.95). This feature was similar to the rotation angle of a beetle's hind wing (see fig. 4(c) in Le *et al*. [45]). The wing was not only twisted in the spanwise direction but was also cambered in the chordwise direction. The variation of the wing camber (which was fitted by 8-term sine and cosine functions) at each wing section in a flapping cycle is shown in figure 7*c*. The camber is defined as the ratio of the height of the mid-chord, denoted by *h* in figure 7*c*, and the chord length, denoted by *c*, at each wing section. The cambers at the seven wing sections from the wing root to the wing tip were less than 20% of the wing chord during both the downstroke and the upstroke, in a manner similar to the chordwise camber in the beetle's hind wing (see fig. 4(d) in Le *et al*. [45]).

### 4.2. Forces produced by flapping wings

**Figure 8.**
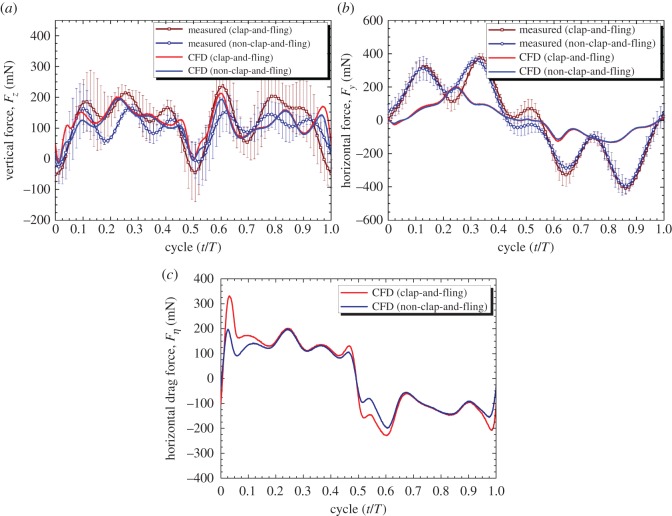
Distribution of (*a*) the vertical force or thrust (*F_z_*), (*b*) horizontal force (*F_y_*) and (*c*) force in the *η*-direction or horizontal drag (*F_η_*) produced by the two flapping-wing systems in a flapping cycle.

The time histories of the measured vertical force (*F_z_*) and horizontal force (*F_y_*) generated by the flapping-wing system with and without implementing the clap-and-fling effect are plotted along with those obtained by the CFD simulation in figure 8*a* and *b*, respectively. The inertial force was not considered in the computational simulation. A study by Truong *et al*. [47] suggested that the inertial force did not affect the average force values but contributed to the change in the time history of forces during the flapping motion. In the current flapping-wing system, the vertical force direction (*z*-direction in figure 4*b*) was perpendicular to the flapping stroke plane (*xy*-plane). Therefore, the inertial force did not significantly affect the time history of the vertical force. As shown in figure 8*a*, the measured time histories of the vertical forces showed similar tendencies to those of the simulated ones even though there were some differences. However, the time histories of the horizontal forces shown in figure 8*b* were strongly affected by the inertial force. As seen in table 1, the average vertical forces (*F_z_*) obtained by the numerical simulation are about 3.2% and 7.5% larger than the measured vertical forces for the cases with and without the clap-and-fling effect, respectively. This proved that the numerical simulation could be used to properly estimate the average forces generated by the FW-MAV.

**Table 1.** Average values of the vertical forces in the *z*-direction (*F_z_*), horizontal forces in the *y*-direction (*F_y_*) and horizontal drag (*F_η_*) in the *η*-direction in a complete flapping cycle. NC&F, non-clap-and-fling; C&F, clap-and-fling.

	average *F_z_* (mN)			average *F_y_* (mN)			average *F_η_* (mN)		
method	NC&F	C&F	enhancement (%)	NC&F	C&F	difference (%)	NC&F	C&F	difference (%)
CFD	108.0	120.4	11.5	2.4	2.4	0.0	3.6	5.1	41.7
measured	100.4^a^	116.7^a^	16.2	2.6^b^	2.9^b^	11.5			
difference (%)	7.5	3.2		−7.7	−17.2				

^a^Standard deviation = 2 mN.

^b^Standard deviation = 0.4 mN.

Table 1 also shows the average horizontal forces in the *y*-direction (*F_y_*) obtained by simulation and measurement. The horizontal forces were less than 3 mN and less than 3% of the vertical forces. The simulated horizontal forces are approximately 17.2% and 7.7% smaller than the measured horizontal forces for the cases with and without the clap-and-fling effect, respectively. This was reasonable given that the minor asymmetric flapping motion generated a small amount of the horizontal force. The results from the CFD simulation indicated that the average horizontal forces in a complete cycle generated by the flapping-wing mechanisms with and without the clap-and-fling effect were the same. However, for the measurement shown in table 1, the difference between the average horizontal forces in the two cases was 11.5%. In reality, this increment was a result of the asymmetric contribution to the average horizontal force of two clap-and-fling mechanisms at the dorsal and ventral stroke reversals, which produced forces in the opposite direction, and was not a result of the clap-and-fling effect. This difference may not significantly affect the horizontal force in the *y*-direction as the average horizontal force in the *y*-direction was small and close to the resolution of the load cell.

The horizontal force in the *y*-direction should not be a drag force in the horizontal plane because of the three-dimensional motion of the flapping wings. Instead, the horizontal drag force should be the force in the *η*-direction (*F_η_*), which is tangential to the wing motion direction as shown in figure 4*b*. However, the measurement was not able to directly capture this horizontal drag force (*F_η_*). Therefore, the time histories and average values of *F_η_* in a flapping cycle were obtained by the CFD simulation and shown in figure 8*c* and table 1, respectively. The average horizontal drag force (*F_η_*) generated by the flapping-wing system with the clap-and-fling effect (5.1 mN) was 41.7% higher than that of the system with a minimized clap-and-fling effect (3.6 mN). This enhancement was not caused by the clap-and-fling effect because of the opposite motions of the wings during the downstroke and upstroke, and the presence of the clap-and-fling mechanisms at both stroke reversals. Instead, in a manner similar to the CFD situation for the horizontal force in the *y*-direction, the difference was caused by the asymmetric contribution of the clap-and-fling effects at the dorsal and ventral stroke reversals.

### 4.3. Contribution of the clap-and-fling effect to force generation

**Figure 9.**
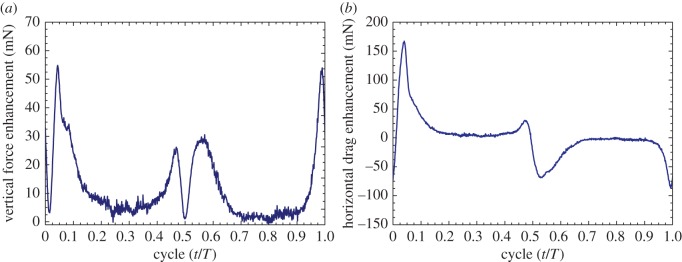
Force enhancement due to the clap-and-fling effect in (*a*) the vertical force or thrust (*F_z_*) and (*b*) force in the *η*-direction or horizontal drag (*F_η_*) in the flapping-wing system.

The average simulated and measured vertical forces in table 1 show that the clap and flings at dorsal and ventral stroke reversals contributed to increases of 11.5% and 16.2%, respectively, when compared with those in the non-clap-and-fling case. Although the average measured forces over cycles are well matched with the estimated forces, the fluctuations in their time histories are not well repeated due to vibratory forces created by flapping wings and mechanism. Therefore, the time histories of the estimated forces are used to investigate contribution of the clap-and-fling effect to the force enhancement. The vertical force enhancement due to the effect of the clap and fling in a flapping cycle from the CFD simulation is plotted in figure 9*a*. As shown in figure 9*a*, the flapping cycles (*t*/*T*) included four force peaks of approximately 0.04, 0.46, 0.57 and 0.98. These peaks exhibited flings at the beginnings of the downstroke and upstroke (*t*/*T* = 0.04 and 0.57, respectively), and claps at the ends of the downstroke and upstroke (*t*/*T* = 0.46 and 0.98, respectively). Thus, the clap as well as the fling contributed to the vertical force enhancement. Table 2 shows the average vertical forces at each quarter of the cycle including the independent clap or fling phase to examine the contribution of each phase to the enhanced vertical force. During the downstroke, the wings flung apart to the translation stage (first quarter) and resulted in an increase of 3.7% in the vertical force. Then, the wings approached each other at the end of clap (second quarter) and contributed to an increase of 1.9% in the vertical force. Hence, the fling and clap at the beginning and the end of downstroke contributed 32.2% and 16.5%, respectively, to the enhanced vertical force in this half flapping cycle. During the upstroke, the fling increased the vertical force by 3.5% (third quarter), while the clap enhanced the vertical force by 2.4% (last quarter). In this half stroke, the contributions of the fling and the clap phases to the enhanced vertical force were 30.4% and 20.9%, respectively. Therefore, in a complete cycle, the clap-and-fling effect improved the vertical force by 11.5%, and the fling phases with a total of 62.6% (from the individual contributions of 32.2% and 30.4%) played a more dominant role than the claps with a total of 37.4% (from the individual contributions of 16.5% and 20.9%) in augmenting the vertical force.

**Table 2.** Contribution of the clap and fling to the average vertical force (*F_z_*) at each stroke period.

	average vertical force, *F_z_* (mN)			
flapping period (*t/T*)	non-clap-and-fling	clap-and-fling	force enhancement (%)	contribution of clap or fling to the enhanced force in 1 cycle (%)
downstroke				
initial fling to mid-stroke (*t*/*T* = 0.012–0.256)	28.6	32.9	3.7	32.2
mid-stroke to the end of clap (*t*/*T* = 0.256–0.499)	27.5	29.7	1.9	16.5
upstroke				
initial fling to mid-stroke (*t*/*T* = 0.499–0.756)	24.0	27.3	3.5	30.4
mid-stroke to the end of clap (*t*/*T* = 0.756–1.012)	27.9	30.5	2.4	20.9
|1 cycle|				
absolute value (*t*/*T* = 0.012–1.012)	108.0	120.4	11.5	100.0

Figure 9*b* plots force enhancements in the *η*-direction or horizontal drag force during a flapping cycle obtained by the CFD simulation. Table 3 shows the details of the contribution of each phase at each quarter of a cycle to the enhanced horizontal drag force (*F_η_*). In a flapping cycle, the fling at the beginning of the downstroke in the first quarter, which is represented by the positive force peak at the time *t*/*T* = 0.04, augmented the horizontal drag force by 6.9%, as shown in table 3. Meanwhile in the second quarter of the cycle, the clap at the end of the downstroke with a small positive force peak at *t*/*T* = 0.48 increased the force by 2.1%. The horizontal drag force during the upstroke was in the negative direction (*t*/*T* = 0.50–1.00). Therefore, its magnitudes were negative as shown in figure 9*b* and table 1. Absolute values of the horizontal drag force were used to investigate the contribution of the clap and fling in this half stroke. During the upstroke, the wings flung apart and subsequently translated to the mid-stroke (third quarter) resulting in a force enhancement of 6.3%. Then, the force was enhanced by 3.1% when the wings translated from the mid-stroke and subsequently clapped at the end of the upstroke (fourth quarter). Thus, the clap-and-fling effect increased the horizontal drag force (*F_η_*) by 18.4% (approx. 1.18 times) during a flapping cycle. In a manner similar to the contribution of the vertical force, most of the enhanced horizontal drag force was a result of the effect of flings at the beginning of the half strokes, which was approximately 71.7%, while the clap contributed to 28.3% in the enhanced horizontal dragforce.

**Table 3.** Contribution of the clap and fling to the average force in the *η*-direction or average horizontal drag force (*F_η_*) at each stroke period.

	average horizontal drag force, *F_η_* (mN)			
flapping period (*t/T*)	non-clap-and-fling	clap-and-fling	force enhancement (%)	contribution of clap-and-fling to enhanced force in 1 cycle (%)
downstroke				
initial fling to mid-stroke (*t*/*T* = 0.012–0.256)	34.6	44.2	6.9	37.5
mid-stroke to the end of clap (*t*/*T* = 0.256–0.499)	26.4	28.5	2.1	11.4
upstroke				
initial fling to mid-stroke (*t*/*T* = 0.499–0.756)	−27.9	−34.6	6.3	34.2
mid-stroke to the end of clap (*t*/*T* = 0.756–1.012)	−29.6	−33.1	3.1	16.9
|1 cycle|				
absolute value (*t*/*T* = 0.012–1.012)	118.5	140.4	18.4	100

**Figure 10.**
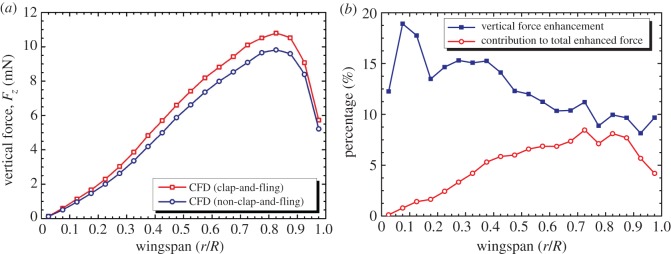
(*a*) Distribution of vertical forces produced by the flapping-wing system with and without considering the effect of the clap and fling and (*b*) enhanced vertical force at each wing section due to the effect of the clap and fling and its contribution to the total enhanced vertical force.

As shown in figure 6*a*, the distance between the left and right wings at the stroke reversals in the model decreased along the wingspan. Therefore, it was necessary to investigate the distribution of the enhanced vertical force along the wingspan. Figure 10*a* plots the vertical forces generated by the flapping-wing system with and without implementing the clap-and-fling effect. Figure 10*b* plots the enhanced vertical force at each wing section along the wingspan and their contribution to the total enhanced vertical force. As observed in figure 10*a*, the magnitude of the vertical force suddenly decreased near the wing tip because of a smaller wing chord length and tip loss effect. As shown in figure 10*b*, the percentage of the vertical force enhancement by the clap-and-fling effect at each wing section was higher near the wing root and was slightly reduced along the wing tip. However, the contribution of the clap-and-fling effect to the total enhancement in the vertical forces was more significant in the outer half of the wing, where the distance between the two wings was closer and thus the clap-and-fling effect wasstronger.

### 4.4. Flow structures

**Figure 11.**
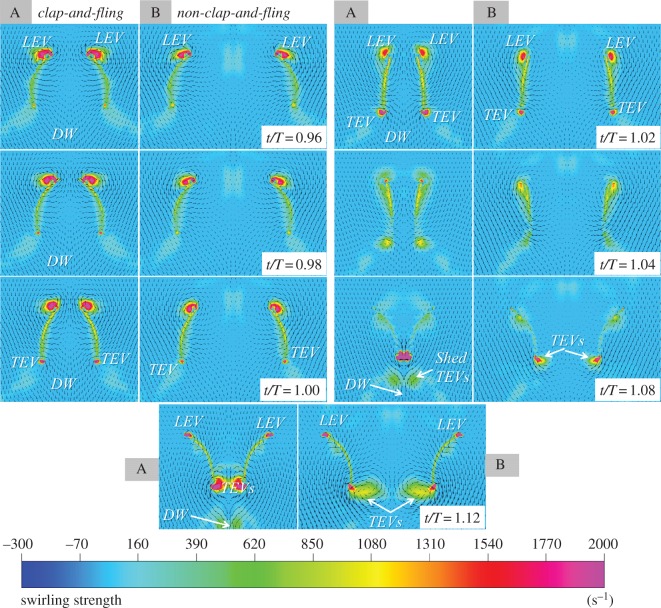
Plots of swirling strength combined with velocity vector at the wingspan of 0.25*R* created by the flapping-wing system with the clap-and-fling effect (case A) and without the clap-and-fling effect (case B). LEV, leading edge vortex; TEV, trailing edge vortex; DW, downwash.

**Figure 12.**
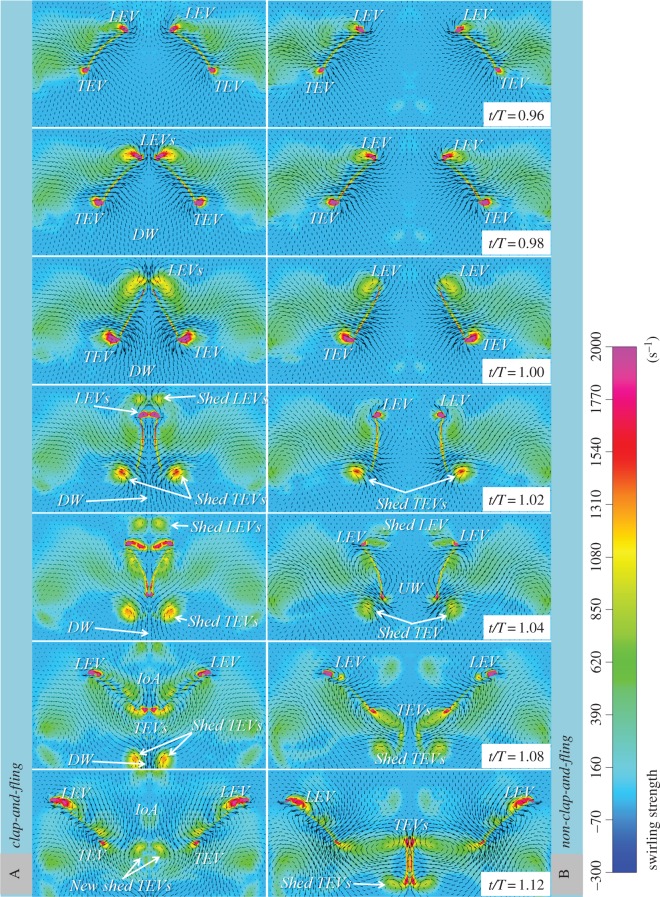
Plots of swirling strength combined with velocity vector at the wingspan of 0.75*R* created by the flapping-wing system with the clap-and-fling effect (case A) and without the clap-and-fling effect (case B). LEV, leading edge vortex; TEV, trailing edge vortex; DW, downwash; IoA, influx of air; UW, upwash.

The study examined the swirling strengths, which are used to visualize vortices, combined with the velocity vectors around the two flapping wings. A distance of 16 mm between the flapping axes was used to implement the clap-and-fling effect (denoted by case A), and a distance of 40 mm between the flapping axes was used in the non-clap-and-fling case (denoted by case B) to examine the role of the clap and fling in augmenting the vertical force generation. As shown in figure 6, the distance between the leading edges of the left and right wings at the end of each stroke was linearly reduced from the wing root to the wing tip. The various wing sections along the wingspan included two wing sections near the wing root and the wing tip with 25% wingspan (0.25*R*) and 75% wingspan (0.75*R*), respectively, which were selected for comparing their flow structures in figures 11 and 12. The sequential images commenced from a non-dimensional time of *t*/*T* = 0.96 to *t*/*T* = 1.12 for both case A and case B, which present the clap-and-fling effect at the dorsal stroke reversal.

Figure 11 shows the snapshots of the swirling strengths and velocity vectors located at 0.25*R* of the two wings. In case A, when the wings approached each other to the end of the upstroke (*t*/*T* = 0.96–1.00), strong leading edge vortices (LEVs) and weak trailing edge vortices (TEVs) that remained attached to the wings were formed. The minimum distance between the leading edges of the two wings at 0.25*R* was approximately 0.5c in case A. The wings rotated about their leading edges to complete the clap of the wings at *t*/*T* = 1.02. At this period of time, the wing chords were nearly parallel but were not in contact with each other. As the wings drew closer to each other from the leading edge to the trailing edge, a downwash developed between the two trailing edges. Then, the wings rotated about their trailing edges to move away from each other (*t*/*T* = 1.04–1.12, fling phase). During this, the LEVs and TEVs were shed and diffused quickly. Subsequently, new weak LEVs and strong TEVs formed and grew in strength (*t*/*T* = 1.08–1.12). As the wings separated from the leading edge to the trailing edge, the airflow rushed into the opening gaps between the leading edges as well as between the trailing edges (*t*/*T* = 1.12). However, the downwash continued to be present beneath the gap between the trailing edges during the fling phase. In case B, strong LEVs and weak TEVs were formed when the wings approached each other from *t*/*T* = 0.96 to *t*/*T* = 1.02. The wings rotated for the next stroke and the vortices were diffused from the leading edges and the trailing edges of the wings (*t*/*T* = 1.04). These characteristics of the airflow were similar to those in case A at the corresponding non-dimensional time period. However, the downwash in case B was not clearly developed in the gap between the trailing edges as that in case A. New LEVs formed and developed when the wings moved apart from each other during the next stroke. Nevertheless, the formed LEVs were weaker than the TEVs in strength (*t*/*T* = 1.08–1.12). The influx of airflow (IoA) in case B, unlike that in case A, was not clearly distinguished when the wings moved away.

The swirling strengths and velocity vectors at 0.75*R* are plotted in figure 12. In case A, LEVs and TEVs were formed when the wings approached each other (*t*/*T* = 0.96–1.00). The minimum distance between the leading edges of the two wings at 0.75*R* was approximately 0.25c. The wings rotated as the leading edges moved apart for the next cycle while the trailing edges came closer (*t*/*T* = 1.02). At this rotation, the vortices were shed from the tips of the wings (leading edges and trailing edges), and new LEVs were generated due to the rotation. When the wings came close to clap, a strong downwash was formed and expelled through the gap between the trailing edges of the wings. The wings commenced the fling when the trailing edges moved apart following the leading edges (*t*/*T* = 1.04–1.12). The motion of each wing broke and divided the previous shed LEV into two parts. A part was shed and rolled upward, and the other part remained attached to the wing surface (*t*/*T* = 1.04). The new LEVs were formed and partly shed (these shed vortices subsequently diffused) at a time (*t*/*T* = 1.04) prior to that when their strength increased (*t*/*T* = 1.08–1.12). Only a part of the air rushed into the opening gap when the wings separated (at *t*/*T* = 1.04) due to the presence of the LEVs shed in the previous stroke. The previously shed LEVs diffused rapidly and the airflow began to invade the opening gap at *t*/*T* = 1.04–1.12. During this time, the TEVs were formed and eventually diffused due to the presence of the mirror wings. Most of the TEVs were mutually exterminated, and therefore the Wagner effect was reduced [9]. It should be noted that the strong downwash between the TEVs shed in the previous stroke appeared throughout the fling period. The airflow structures in case B (non-clap-and-fling case) were quite similar to those in case A for the same instant in time, with the exception of a few major differences. First, the downwash formed but was not clear when the wings approached and separated from each other (case B, *t*/*T* = 0.96–1.12). As the wings moved away from each other, the IoA in the opening gap was from both the leading edges and also from the trailing edges between the wings due to the presence of trailing edge circulation. Additionally, the motion of the previous shed TEVs in this case was slower than that in case A (*t*/*T* = 1.04–1.12). Hence, the presence of the downwash when the wings drew close to each other and then moved apart in case A (the case with the clap-and-fling effect), was the main source of the augmented vertical force. Furthermore, the IoA in the low-pressure region between the wings from the leading edges also significantly contributed to enhance the vertical force.

### 4.5. Discussion

This study was inspired by the flight performance of the rhinoceros beetle. Hence, in this study, the clap-and-fling effect was implemented in a two-winged FW-MAV for the first time. This involved designing the flapping mechanism, which combined the 4-bar linkage and pulley–string mechanism, such that it was able to flap the wings with a high flapping amplitude of approximately 192°, as shown in figure 7*a*. However, only the wing tips approximately touched at this flapping amplitude (figure 6*a*) exhibiting near-clap-and-fling case due to the limitation of the mechanism design [37]. Moreover, the wings were flexible during the flapping motion creating a chordwise camber and spanwise twist (figure 7*b*,*c*). During the clap and fling, the effect of wing flexibility allowed the fling to act like a peel, such that the wings separated along the wing chord from the leading edge to the trailing edge, and then clapped in a manner similar to a reverse peel [12,17,58]. Thus, the clap and fling in the FW-MAV developed in this study could be regarded as a near-clap-and-peel mechanism.

Both experiments and simulations were used to investigate the effect of the near clap and peel on the force enhancement at a relatively high Reynolds number of approximately 15 000. The measurement results showed that the clap and flings enhanced the vertical force by 16.2%, while the simulation indicated that the clap and flings increased the vertical force by 11.5%. This could be due to the slight difference between the fitted and the measured flapping angle at the end of each stroke (figure 7*a*). Although a fitted function consisting of eight terms was used, the peak-to-peak value of the fitted flapping angle was still approximately 3° smaller than the measured flapping angle. Therefore, the distance between the two wings at the ends of the strokes along the wingspan in the simulation exceeded that in the measurement. This could have reduced the effect of the clap and fling on the force generation in the simulation [36].

The contribution of each phase to the augmented forces was considered in the developed FW-MAV. Several previous studies on insects and robotic wings at low Reynolds numbers (*Re* < 1000) suggested that both clap and fling phases contribute to the force enhancement [11,12,23,36,37,58]. At a high Reynolds number range (*Re* > 10 000), the effect of clap was not considered [33] or its contribution to the force enhancement was not significant [42]. However, the study results indicated that the clap as well as the fling contributed significantly to the vertical force enhancement in a relatively high Reynolds number (approx. 15 000) environment. Owing to the presence of the mirror wing during the clap and fling, the airflow beneath the trailing edges was pushed downward throughout the period of clap and fling (figure 12*a*, *t*/*T* = 0.96–1.12) providing an additional vertical force. The behaviour of this downwash differed from previous studies, in which the downwash was observed when the wings clapped together only [26,28,36,58]. In addition to fling phase, the IoA in the low-pressure region between the wings caused another increase in the vertical force. Therefore, the fling and the clap contributed to approximately 62.6% and 37.4% of the vertical force enhancement, respectively. There were two clap-and-fling mechanisms at the dorsal and ventral stroke reversals in the developed FW-MAV. As shown in table 2, the dorsal clap and fling at the end of the upstroke and beginning of the downstroke made a contribution to the enhanced vertical force by 53.1%, while the ventral clap and fling at the end of the downstroke and the beginning of the upstroke contributed to 46.9% of the vertical force enhancement. This slight asymmetric contribution could be the result of the asymmetry in the flapping motion, where the distance between the left and right wings at the dorsal stroke reversal was less than that at the ventral stroke reversal.

The analysis of the effect of clap and fling at each portion in the time history of the horizontal drag force (*F_η_*) in a complete flapping cycle indicated that the clap and flings influenced and increased the horizontal drag by approximately 1.18 times that of the horizontal drag force generated by the flapping-wing system without the clap-and-fling mechanisms. This result contradicted the results of previous studies [12], which suggested that the enhancements in the drag force by the rigid fling and the flexible fling were approximately 10 and 5 times that of the drag force produced without the clap-and-fling effect. This could be due to the fact that the two wings in the model in this study did not physically touch each other at each stroke reversal. Additionally, the wings were operated at a relatively higher Reynolds number (approx. 15 000) than those in a previous study by Miller & Peskin [12], where the wings were operated at a Reynolds number of only 10. Another study by Miller & Peskin [11] showed that the enhanced drag force due to the effect of clap and fling was smaller for a high Reynolds number than for a low Reynolds number.

**Figure 13.**
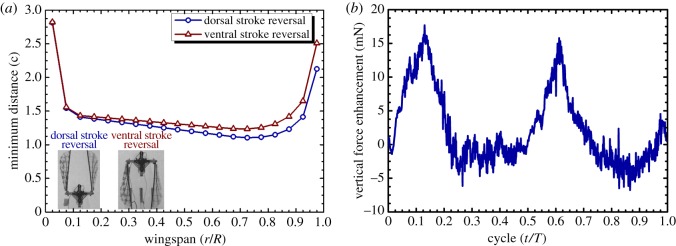
(*a*) Minimum distance at the stroke reversals between two wings along the wingspan in the flapping-wing mechanism in the case of *d *= 40 mm (denoted by the non-clap-and-fling case) and (*b*) vertical force enhancement due to the presence of the clap-and-fling in the non-clap-and-fling case compared with the single-wing case.

A two-dimensional study by Sun & Yu [36] at a low Reynolds number of 17 indicated that the effect of the clap and fling was very small when the distance between two aerofoils was approximately 1c. Figure 13*a* plots the minimum distances along the wingspan between two wings at the stroke reversals in the flapping-wing system with the flapping axes distance (*d *= 40 mm), which is denoted by the non-clap-and-fling case. The figure shows that all the distances exceeded 1.1c at the dorsal stroke reversal (*t*/*T* = 0.00–0.50) and 1.2c at the ventral stroke reversal (*t*/*T* = 0.50–1.00). A single-winged case was simulated and used for comparing the generation of vertical forces to verify whether or not the clap-and-fling effect was still present in this case. The results revealed that the average vertical force generated by the non-clap-and-fling case (108.0 mN) was only approximately 2.0% higher than that of the single-winged case (105.9 mN). As shown in figure 13*b*, most of the enhanced vertical force was from the fling effect at the beginning of each stroke. Hence, this result agreed with that in the study by Sun & Yu [36], although the effect of the clap and fling was not fully removed in the non-clap-and-fling case. Therefore, the result in the study by Sun & Yu [36] could be also applied to relatively high Reynolds number environments.

## 5. Conclusion

This study proposed a hovering insect-like two-winged FW-MAV with high flapping amplitude generation, which presented a clap and fling at each stroke reversal for the first time. Numerical and experimental approaches were employed to investigate the effect of the clap-and-fling mechanism on the force generation. The estimated forces from the CFD model based on the three-dimensional deformable wing kinematics agreed well with the forces measured by a 6-axis load cell, with differences of approximately 7.5% and 7.7% for vertical (*F_z_*) and horizontal (*F_y_*) forces, respectively. From the measurement, the clap and flings at both stroke reversals augmented the average vertical force by approximately 16.2% when compared with that in the case in which the minimum distance between the two wings was extended to 1c. The CFD results indicated that the clap and flings enhanced the vertical force by approximately 11.5% and horizontal drag force (*F_η_*) by approximately 18.4%. Analyses of the force generation at each quarter of a flapping cycle revealed that approximately 62.6% of the enhanced vertical force was attributed to the fling effect at the beginning of the strokes, while the claps at the ends of strokes contributed to the vertical force enhancement by approximately 37.4%. With respect to the enhanced horizontal drag force, the flings contributed to approximately 71.7% of the force increase and the claps contributed to 28.3% of the force enhancement. The enhanced force could be explained by the airflow structures. That is, a strong downward jet expelled from the trailing edges during the fling as well as the clap at each stroke reversal is a mechanism of the force enhancement. Additionally, the influx of air into the low-pressure region between the wings from the leading edges during the fling phases also significantly contributed to the enhanced force. Thus, the clap and flings played a significant role in improving the vertical force in the hovering insect-like two-winged FW-MAV developed in this study. In a relatively high Reynolds number environment, it was also revealed that the effect of the clap and fling was insignificant when the minimum distance between the two wings exceeded 1.2c.

## Data accessibility

Data used for figures 7–10 and figure 13 in this paper are available from the Dryad Digital Repository: http://dx.doi.org/10.5061/dryad.28713 [59].
